# High-Frequency rTMS of the Motor Cortex Modulates Cerebellar and Widespread Activity as Revealed by SVM

**DOI:** 10.3389/fnins.2020.00186

**Published:** 2020-03-19

**Authors:** Jue Wang, Xin-Ping Deng, Yun-Ying Wu, Xiao-Long Li, Zi-Jian Feng, Hong-Xiao Wang, Ying Jing, Na Zhao, Yu-Feng Zang, Jian Zhang

**Affiliations:** ^1^School of Psychology, Shanghai University of Sport, Shanghai, China; ^2^Institute of Psychological Sciences, Hangzhou Normal University, Hangzhou, China; ^3^Zhejiang Key Laboratory for Research in Assessment of Cognitive Impairments, Hangzhou, China; ^4^Center for Cognition and Brain Disorders, The Affiliated Hospital of Hangzhou Normal University, Hangzhou, China

**Keywords:** rTMS, fMRI-guided navigation, motor cortex, cerebellum, resting-state fMRI

## Abstract

Functional magnetic resonance imaging (fMRI) studies have shown that the effect of repetitive transcranial magnetic stimulation (rTMS) can induce changes in remote brain regions. In the stimulated regions, low-frequency (≤1 Hz) rTMS induces inhibitory effects, while high-frequency (≥5 Hz) stimulation induces excitatory effects. However, these stereotypical effects arising from low- and high-frequency stimulation are based on measurements of motor evoked potentials (MEPs) induced by pulsed stimulation. To test the effects of rTMS on remote brain regions, the current study recruited 31 young healthy adults who participated in three rTMS sessions (10 Hz high frequency, 1 Hz low frequency, and sham) on three separate days. The stimulation target was based on individual fMRI activation in the motor cortex evoked by a finger movement task. Pre- and post-rTMS resting-state fMRI (RS-fMRI) were acquired. Regional homogeneity (ReHo) and degree centrality (DC) were calculated to measure the local and global connectivity, respectively. Compared with the sham session, high-frequency (10 Hz) rTMS significantly increased ReHo and DC in the right cerebellum, while low-frequency (1 Hz) stimulation did not significantly alter ReHo or DC. Then, using a newly developed PAIR support vector machine (SVM) method, we achieved accuracy of 93.18–97.24% by split-half validation for pairwise comparisons between conditions for ReHo or DC. While the univariate analyses suggest that high-frequency rTMS of the left motor cortex could affect distant brain activity in the right cerebellum, the multivariate SVM results suggest that both high- and low-frequency rTMS significantly modulated widespread brain activity. The current findings are useful for increasing the understanding of the mechanisms of rTMS, as well as guiding precise individualized rTMS treatment of movement disorders.

## Introduction

Repetitive transcranial magnetic stimulation (rTMS) is a safe and non-invasive technique for the treatment of brain diseases. It is widely believed that low-frequency (≤1 Hz) rTMS exhibits inhibitory effects and high-frequency (≥5 Hz) rTMS exhibits excitatory effects on brain activity (Lefaucheur, 2019). However, these conclusions are primarily based on measurements of the amplitude of motor evoked potentials (MEPs) elicited by pulsed TMS of the primary motor cortex. In fact, the modulatory effects of rTMS on brain activity are much more complicated.

Resting-state functional magnetic resonance imaging (RS-fMRI) is increasingly being used to detect TMS-induced brain activity at the network level. Many studies using this technique have found that rTMS modulates brain networks or functional connectivity (FC) (Eldaief et al., 2011; Chen et al., 2013; Halko et al., 2014; Nettekoven et al., 2014, 2015; Wang et al., 2014; Watanabe et al., 2014; Andoh et al., 2015; Cocchi et al., 2015, 2016; Ji et al., 2017). While RS-fMRI studies into FC have increased our understanding of the complex mechanisms of the modulatory effects of rTMS on brain activity, there are two limitations for analysis of FC or networks. First, these analyses can only reveal the relationships between brain activity in distinct regions. For example, one study found that rTMS modulated the FC between the lateral parietal cortex with the hippocampus (Wang et al., 2014), but it is not clear whether the local activity in the hippocampus was modulated. An alteration in FC does not indicate a change in brain activity in a specific region. FC is probably a “bridge” to deliver the stimulus to the hippocampus. The second limitation is that there are too many options for the configuration of network or FC analysis. For example, the most popular seed-based FC analysis has countless options for the location of the seed region of interest (ROI). This makes it difficult to compare the results between different studies using network or FC analysis.

In contrast to network or FC analyses that compare activity between distributed brain regions, there are a few RS-fMRI metrics that reflect spontaneous local activity, such as regional homogeneity (ReHo) (Zang et al., 2004). This measures the local synchronization of the nearest neighboring voxels (e.g., 7, 19, or 27 voxels). Another metric, degree centrality (DC), measures the strength of the connectivity of one voxel compared with all other voxels in the brain (Buckner et al., 2009). DC is one of the least computationally consuming metrics of graph theory and can be easily accomplished at the voxel level. Combined measurements of ReHo and DC could reflect both local and global connectivity of a specific voxel. ReHo and DC are metrics of “voxel-level whole-brain” analysis (Zang et al., 2015). In addition, there are far fewer options for their analysis parameters than other FC methods (such as the excess of options for seed selection in seed-based FC analysis). These characteristics of ReHo and DC render them more suitable for coordinate-based meta-analysis (Zang et al., 2015) and are further helpful for precise localization of abnormal activity.

Typical univariate neuroimaging analyses compare differences between groups in a voxel-wise or region-wise manner. Multivariate analyses can be applied using machine learning classification techniques, such as support vector machine (SVM). In most studies where it is applied, machine learning is used for differentiating between two independent groups, for example, comparing patient group to healthy control. Sometimes, machine learning methods are used to differentiate two conditions within a group. Borrowing from the concept of paired *t* tests, Zhou et al. (2017) proposed a PAIR method for SVM. Compared with conventional UNPAIR SVM (i.e., taking two within-group conditions as independent conditions), PAIR SVM yielded similar performance when applied to an RS-fMRI dataset with two conditions (eyes closed vs. eyes open), but better performance when validating in a completely new dataset (Zhou et al., 2017), suggesting that PAIR SVM could be better generalized.

The motor cortex is one of the most frequently reported stimulation targets for rTMS modulation in both healthy populations (Hartwigsen and Siebner, 2015; Cona et al., 2017) and those with brain disorders including movement disorders (Wagle Shukla et al., 2016; Brabenec et al., 2019), stroke rehabilitation (Ludemann-Podubecka et al., 2016; Lee et al., 2019), and other disorders (Siebner et al., 2003; Odorfer et al., 2019; Pei et al., 2019; Zhang et al., 2019). Some of these studies performed RS-fMRI before and after modulation and analyzed the network changes. However, there is large variation in the analytical methods applied from study to study. These included voxel-to-voxel based dynamic FC (Zhang et al., 2019), graph theory using 24 ROIs (Lee et al., 2019), whole-brain graph theory (Pei et al., 2019), and seed-based FC (Brabenec et al., 2019). While it could be concluded that rTMS of the motor cortex modulates the motor network, such a conclusion appears too general since it is difficult to identify which specific brain areas are modulated.

The current study aimed to investigate the modulatory effects of rTMS on specific brain areas by measuring local RS-fMRI metrics. We compared low-frequency (1 Hz) and high-frequency (10 Hz) rTMS with a sham condition. For precise and individualized localization of rTMS, self-initiated finger movement task was performed and the fMRI activation peak voxel in the motor cortex was taken as the stimulation target for each individual. We hypothesized that local spontaneous activity in motor-related subcortical regions could be modulated. In addition to univariate statistical analyses (ANOVA and *t* tests), we used PAIR SVM (Zhou et al., 2017) to differentiate between rTMS conditions.

## Materials and Methods

### Participants

Thirty-three healthy right-handed participants were recruited through an online advertisement. Two participants were excluded, one because head motion exceeded 2 mm in translation or 2° in rotation in any direction, and one because there was no task-related activation in the fMRI. A total of 31 participants were included in the final analysis (23 females, mean age ± SD: 23 ± 2.8 years). All participants met the inclusion criteria of no history of neuropsychiatric disorders or head trauma, no substance abuse, and no psychiatric disorders. The whole study was approved by the Ethics Committee of the Center for Cognition and Brain Disorders (CCBD) at Hangzhou Normal University (HZNU). Informed consent was obtained from each participant before the first scanning session.

### Experimental Design

Our experiment consisted of a within-subject, single-blinded, and placebo-controlled design. Each participant received three sessions of rTMS intervention (one each of 10 Hz, 1 Hz, and sham stimulation) across three separate days with an interval of more than 1 week between each session. The order of the conditions was balanced across participants. Participants underwent an RS-fMRI scan session, a task fMRI session, and a 3D-T1 session before rTMS. They then received an rTMS intervention in the TMS room near the MRI room. Immediately (less than 30 min) after that, both RS-fMRI and task fMRI were scanned again.

During RS-fMRI scanning, participants were asked to keep their eyes closed, relax, remain as motionless as possible, not think of anything in particular, and not fall asleep.

**FIGURE 1:**
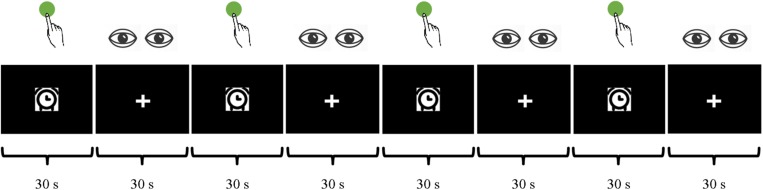
Finger tapping, a block design task with a self-paced rhythm at around 2 s, during a 4-min MRI scan.

During the task fMRI session, participants were asked to perform a 4-min block design task consisting of finger tapping. For the finger tapping blocks, participants were asked to press a button with their right index finger with a self-paced rhythm about every 2 s when a picture of clock appeared in the center of the screen. The picture remained visible for the whole 30 s of the block (Figure 1). For the 30-s rest blocks, participants were asked to relax with their eyes fixed on a cross in the center of the screen.

### MRI Data Acquisition

MRI data were acquired on a 3T scanner (MR-750, GE Medical Systems, Milwaukee, WI, United States) at the CCBD of HZNU. The fMRI scanning sessions included an 8-min RS-fMRI session and a 4-min task session with the following parameters: repetition time (TR) = 2000 ms, echo time (TE) = 30 ms, flip angle (FA) = 90°, 43 slices with no gaps, matrix = 64 × 64, field of view (FOV) = 220 mm × 220 mm, acquisition voxel size = 3.44 mm × 3.44 mm × 3.2 mm. A high-resolution T1 anatomical image was obtained (176 sagittal slices, thickness = 1 mm, TR = 8.1 ms, TE = 3.1 ms, FA = 8°, FOV = 250 × 250 mm).

### Data Analysis on Pre-rTMS MRI

The pre-rTMS task fMRI data and T1 image acquired on the first day were used to localize the stimulation target for each individual. Statistical parametric mapping 12 (SPM12^1^) was used for subject-level activation analysis (high-pass filtering, >1/128 Hz, was selected in “fMRI Model specification”) after preprocessing, which included slice timing correction, head motion correction, co-registering the functional images to T1 image, and then spatial smoothing with a Gaussian kernel of 6 mm full width at half maxima (FWHM). Finally, the individual activation map was generated using a linear general model. Then, for each participant, the individual peak activation voxel around “hand knob/M1” (Yousry et al., 1997) was identified as the individualized rTMS stimulation target. The motor cortex was successfully activated in 31 participants (Table 1).

**TABLE 1:** Peak voxels of finger tapping activation in the motor cortex.

**Subject**	**Brodmann**	**Coordinate**			***t* value**	***p* value**
**ID**	**area**	**(*x y z*)**				
Sub001	4	-44	-15	58	6.25	<0.001
Sub002	6	-54	-6	50	6.22	<0.001
Sub003	4	-36	-19	59	5.84	<0.001
Sub004	4	-32	-20	56	3.91	<0.001
Sub005	4	-42	-16	62	8.89	<0.001
Sub006	6	-43	-8	63	5.05	<0.001
Sub007	6	-42	-14	49	8.36	<0.001
Sub008	6	-51	-9	51	9.21	<0.001
Sub009	6	-43	-14	58	3.82	<0.001
Sub010	6	-50	-6	49	6.40	<0.001
Sub011	6	-50	4	47	6.34	<0.001
Sub012	4	-42	-15	56	8.29	<0.001
Sub013	6	-57	0	44	4.44	<0.001
Sub014	6	-42	-12	57	10.1	<0.001
Sub015	6	-36	-15	57	8.58	<0.001
Sub016	6	-30	-24	60	2.78	<0.01
Sub017	6	-39	-9	54	5.91	<0.001
Sub018	4	-36	-25	61	8.59	<0.001
Sub019	4	-48	-13	58	5.97	<0.001
Sub020	6	-51	-7	51	9.44	<0.001
Sub021	4	-45	-15	56	5.55	<0.001
Sub022	4	-36	-23	54	8.45	<0.001
Sub023	4	-54	-7	42	9.90	<0.001
Sub024	6	-45	-3	47	3.32	<0.01
Sub025	4	-55	-3	42	7.64	<0.001
Sub026	4	-43	-15	56	5.26	<0.001
Sub027	6	-51	-5	50	6.95	<0.001
Sub028	6	-51	-6	48	6.92	<0.001
Sub029	6	-50	0	48	11.28	<0.001
Sub030	6	-57	0	44	6.93	<0.001
Sub031	3	-32	-27	57	8.52	<0.001
Mean		-45	-10	53		

### fMRI-Navigated rTMS

The individual activation map was loaded into BrainSight TMS navigation system (Rogue Research, Montreal, Canada) for fMRI-guided rTMS intervention. TMS (Magstim Rapid^2^, Magstim Co., Whitland, United Kingdom) was applied with a figure-of-8 coil (diameter = 70 mm). Surface electromyogram (EMG) leads were placed over the right abductor pollicis brevis (APB) muscle. Participants sat in a cozy chair with both arms relaxed on their thighs. Full muscle relaxation was confirmed through visual observation and EMG monitoring. The coil (toward forehead) was firstly placed over the left primary motor area (M1, hand knob) at an angle of 45° from the coronal midline for measuring the MEPs in the target muscle during rTMS sessions. To determine the hotspot, the coil was moved by distances of 0.5 cm around the hand knob area. The resting motor threshold (RMT) was quantified as the lowest intensity that evoked a response (>50 μV) in more than 5 of 10 consecutive trials.

For each stimulation day, 1800 pulses (intensity of 100% RMT, duration 30 min) were delivered (Eldaief et al., 2011). For the low-frequency (1 Hz) stimulation, the pulses were delivered continuously for 1800 s. For the high-frequency (10 Hz) stimulation, the pulses were delivered with 60 trains of stimulation each lasting 3 s, with rest intervals of 27 s in between (total duration: 1800 s). For the sham stimulation, the coil was tilted 90° off the scalp with one wing touching the scalp (Lisanby et al., 2001). Sham stimulation was randomly assigned at 1 Hz with half of participants and 10 Hz with the other half. No side effects of rTMS occurred in the current study.

### Data Preprocessing for Group-Level Comparisons of Pre- and Post-rTMS fMRI

The RS-fMRI data preprocessing was conducted using DPABI_V4.0^2^ software (Yan et al., 2016) and included (1) discarding the first 10 volumes to allow the signal to reach equilibrium and the subjects to adapt to the scanning noise, (2) correcting for the acquisition time delay between slices, (3) rigid-body realignment for estimation and correction of motion displacement, (4) co-registering the functional images to the T1 image, (5) normalization to MNI space using the EPI template in SPM12, (6) regressing out 24 head-motion parameters (Yan et al., 2013), (7) removing the linear trend, and (8) band-pass filtering (0.01–0.08 Hz). After the preprocessing, ReHo (Zang et al., 2004) and DC were calculated. For the ReHo calculation, the Kendall concordance coefficient was calculated for the time courses of seven neighboring voxels. We did not use the conventional 27 neighboring voxels because we were interested in subcortical areas, most of which have a small volume. For DC calculation, a correlation coefficient *r* > 0.25 was set as the threshold, and the negative connections were excluded when calculating weighted DC maps because of their ambiguous interpretation (Murphy et al., 2009; Weissenbacher et al., 2009; Wang et al., 2011). A predefined gray matter mask provided by SPM12^3^, with tissue probability >20%, was used to restrict the DC calculation within the gray matter (Zuo et al., 2012). Spatial smoothing with a Gaussian kernel of 6 mm FWHM was then applied to the mReHo (ReHo value of each voxel divided by the mean ReHo value of the whole gray matter mask) and weighted positive mDC (weighted positive DC value of each voxel divided by the mean weighted positive DC value of the whole gray matter mask) maps.

### Statistical Analysis

#### Univariate Analysis

One-way ANOVAs were conducted on the ReHo change and DC change (post- minus pre-rTMS) to explore differences between the three stimulation conditions (Low, 1 Hz; High, 10 Hz; and Sham) within the predefined gray matter mask. The ANOVA *F* maps were corrected using Gaussian random field (GRF) correction (single voxel *p* < 0.001, cluster level *p* < 0.05). The ReHo and DC values of the peak voxels in the surviving clusters were extracted, and then were entered into SPSS (v20^4^) for further analysis. Paired *t* tests were performed between stimulation conditions (High vs. Low, High vs. Sham, and Low vs. Sham).

#### Multivariate Analyses Using SVM

##### Dimensionality reduction

The *F* map was thresholded at *p* < 0.05 (uncorrected) to generate a mask for the feature extraction of each condition (High, Low, and Sham) and each metric (the ReHo and DC value of post- minus pre-rTMS for each condition). Thus, 4267 voxels from the gray matter mask (67,541 voxels) were used for SVM analysis.

##### Grouping for PAIR SVM

PAIR SVM is a new method for differentiating two conditions in the context of a within-group design. It has been found to show better generalization performance in an independent dataset than using the UNPAIR method (Zhou et al., 2017) and was therefore implemented in the current study. To explain, we take the comparison of ReHo change (ReHo value of post- minus pre-rTMS) between High vs. Sham as an example. The 31 participants were randomly divided into groups A and B (*n* = 15 and 16, respectively). Group A was assigned to be the “High minus Sham” group (labeled “ + 1”) and group B was assigned to be the “Sham minus High” group (labeled “−1”).

#### SVM Classification

The linear kernel was used to extract the weight for each feature (Fu et al., 2008). The sequential minimal optimization (SMO) algorithm was used to handle the very large training datasets with high speed (Platt, 1998). For optimal generalization, we used split-half cross-validation, which is more stringent than other methods such as n-fold or leave-one-out validation. Half of the dataset (15 or 16 random samples from group A and group B, respectively) was randomly selected to train the SVM model, and the remaining half was used as test data. We then obtained the classification accuracy and corresponding weighted contribution vector. These calculations were repeated 100 times, and then the mean accuracy and the mean weighted vector were obtained.

## Results

**FIGURE 2:**
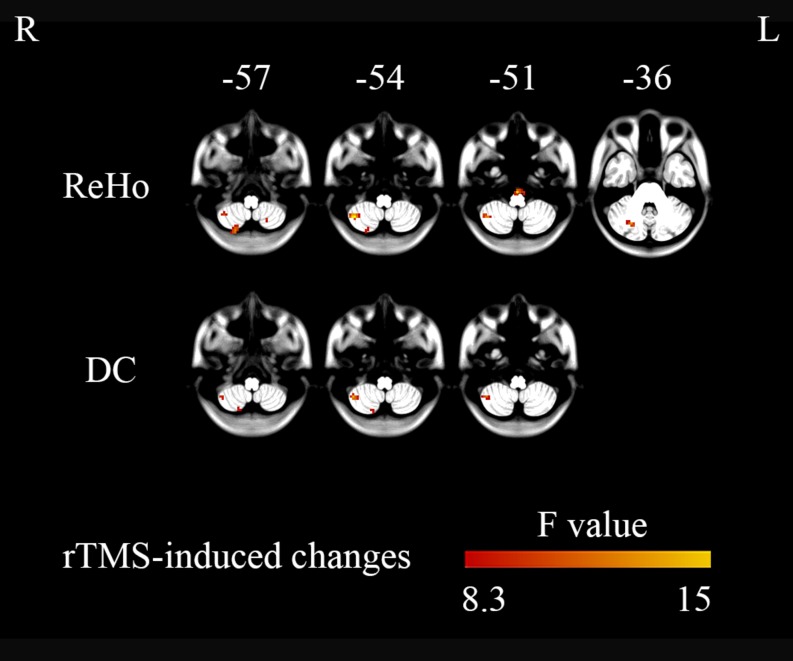
Three-level (1 Hz/low frequency, 10 Hz/high frequency, and sham) one-way ANOVA on changes in brain activity (post- minus pre-rTMS). The right cerebellum showed significant differences between the three stimulation conditions (GRF correction, single voxel *p* < 0.001, cluster level *p* < 0.05).

**FIGURE 3:**
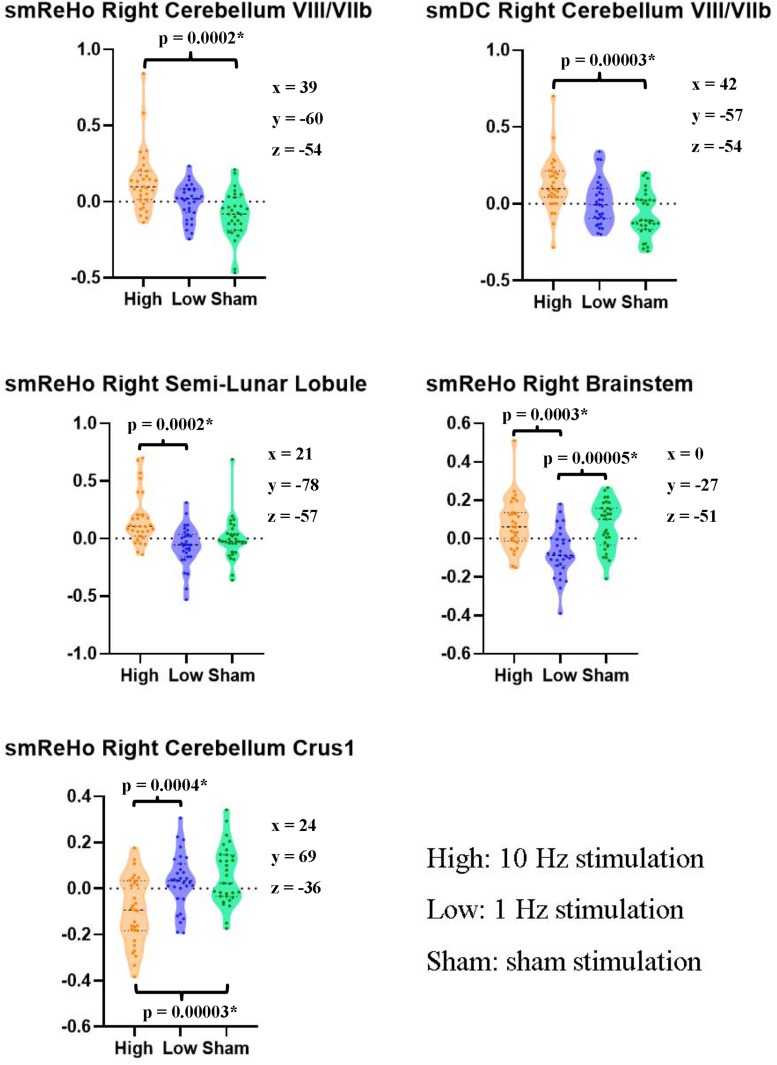
Pairwise paired *t* tests between stimulation conditions on the ReHo and DC value of the peak voxel of *F* maps in the right cerebellum. The coordinates of the peak voxels were the same as Table 2. *Still significant after Bonferroni correction of 0.05/15 = 0.0033.

**FIGURE 4:**
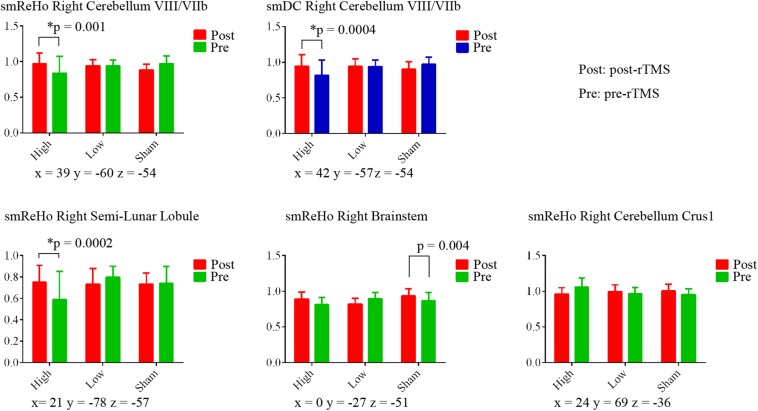
Paired *t* tests between post- and pre-rTMS on ReHo and DC value of the peak voxel of *F* maps in the right cerebellum. The coordinates of the peak voxels were the same as Table 2. *Still significant after Bonferroni correction of 0.05/15 = 0.0033.

ANOVAs showed significant effects between the three conditions (Low, High, and Sham) in the right cerebellum VIII extending to VIIb for both ReHo and DC (Figure 2 and Table 2). Paired *t* tests showed that the high-frequency stimulation (10 Hz) revealed a significantly larger change than the sham stimulation (mean ± SD = 0.22 ± 0.29, *t* = 4.22, *p* = 0.0002, Bonferroni corrected, i.e., 0.05/15 = 0.0033; Figure 3 and Table 3) for ReHo, and was significantly larger change than the sham stimulation (mean ± SD = 0.20 ± 0.22, *t* = 4.95, *p* = 0.00003, Bonferroni corrected, i.e., 0.05/15 = 0.0033; Figure 3 and Table 3) for DC. The results of ReHo and DC were similar, while with a bit slightly more regions for ReHo. Post- vs. pre-rTMS effects of each stimulation condition is shown in Figure 4.

**TABLE 2:** Alterations in activity (post- minus pre-rTMS) in different brain regions for the High, Low, and Sham stimulation conditions from one-way ANOVAs.

**Brain region**	**MNI (*x y z*)**			**Cluster size (mm^3^)**	***F* value**	**Peak voxel *p* value**
**ReHo**						
Right Inferior Semi-Lunar Lobule	21	-78	-57	621	11.571	<0.001
Left Inferior Semi-Lunar Lobule	-21	-63	-57	351	9.29	<0.001
Right Cerebellum VIII/VIIb	39	-60	-54	864	15.02	<0.001
Right Brainstem	0	-27	-51	459	14.18	<0.001
Right Cerebellum Crus1	24	-69	-36	459	12.26	<0.001
**DC**						
Right Inferior Semi-Lunar Lobule	18	-75	-57	405	8.86	<0.001
Right Cerebellum VIII/VIIb	42	-57	-54	729	12.91	<0.001

*ReHo, regional homogeneity; DC, degree centrality; MNI, Montreal Neurological Institute.*

**TABLE 3:** Differences between stimulation conditions in the right cerebellum (paired *t* tests).

	**Mean ± SD**	***t* value**	***p* value**
**ReHo (Right Cerebellum VIII/VIIb)**			
High vs. Low	0.13 ± 0.24	3.13	0.004
High vs. Sham	0.22 ± 0.29	4.22	0.0002^∗^
Low vs. Sham	0.09 ± 0.19	2.48	0.019
**DC (Right Cerebellum VIII/VIIb)**			
High vs. Low	0.12 ± 0.23	2.96	0.006
High vs. Sham	0.20 ± 0.22	4.95	0.00003^∗^
Low vs. Sham	0.08 ± 0.17	2.56	0.016
**ReHo (Right Semi-Lunar Lobule)**			
High vs. Low	0.23 ± 0.30	4.16	0.0002^∗^
High vs. Sham	0.17 ± 0.33	2.80	0.009
Low vs. Sham	−0.06 ± 0.28	−1.17	0.253
**ReHo (Right Brainstem)**			
High vs. Low	0.15 ± 0.21	4.11	0.0003^∗^
High vs. Sham	0.01 ± 0.20	0.25	0.805
Low vs. Sham	−0.14 ± 0.17	−4.73	0.00005^∗^
**ReHo (Right Cerebellum Crus1)**			
High vs. Low	−0.13 ± 0.18	−3.98	0.0004^∗^
High vs. Sham	−0.15 ± 0.17	−4.87	0.00003^∗^
Low vs. Sham	0.03 ± 0.19	−0.74	0.463

*^∗^Surviving Bonferroni correction of 0.05/15 = 0.0033. SD, standard deviation; ReHo, regional homogeneity; DC, degree centrality.*

**FIGURE 5:**
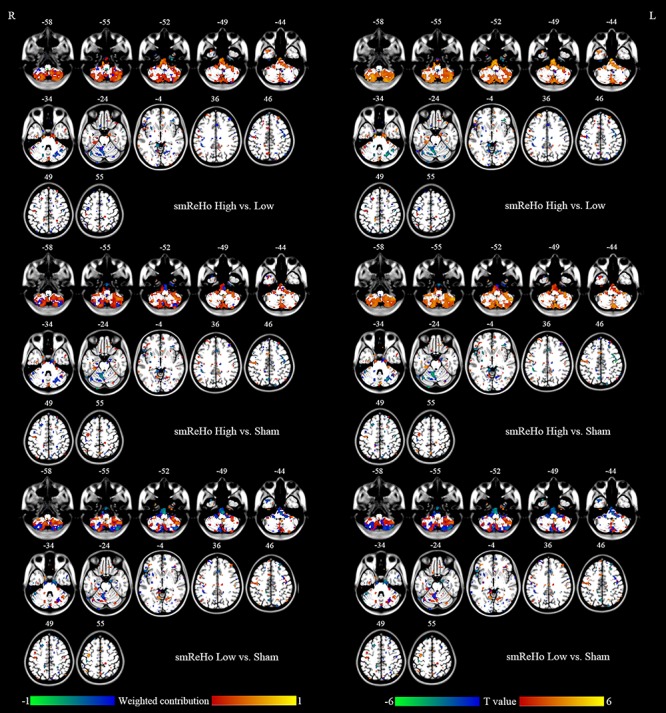
Left column: Brain regions that showed different contributions between stimulation conditions identified by ReHo maps within the mask of uncorrected *F* map of ANOVA. Right column: Paired *t* tests between stimulation conditions on ReHo maps within the mask of uncorrected *F* map of ANOVA.

**FIGURE 6:**
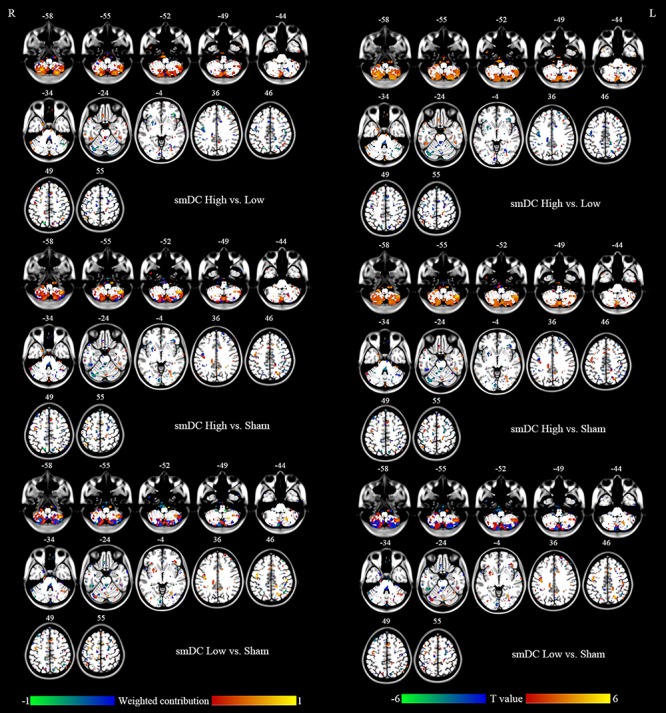
Left column: Brain regions that showed different contributions between stimulation conditions identified by DC maps within the mask of uncorrected *F* map of ANOVA. Right column: Paired *t* tests between stimulation conditions on DC maps within the mask of uncorrected *F* map of ANOVA.

For the SVM, the classification accuracy of the split-half validation ranged from 93.18 to 97.24%, specifically: 94.7% (ReHo) and 93.18% (DC) for the High vs. Low condition, 95.95% (ReHo) and 94.32% (DC) for the High vs. Sham, and 97.24% (ReHo) and 95.57% (DC) for the Low vs. Sham (Table 4). The spatial pattern of voxel-level contribution for discriminative results was very similar to the spatial patterns of paired *t* tests between stimulation conditions (ReHo: Figure 5, DC: Figure 6).

**TABLE 4:** The mean classification accuracy of split-half validation and the area under the curve (AUC) of the receiver operating characteristic (ROC) of the peak voxels of the *F* map of ReHo and DC for pairwise comparison between rTMS conditions.

**PAIR SVM**			
	**High**	**Low**	**Sham**
**ReHo**			
High		94.70%	95.95%
Low			97.24%
Sham			
**DC**			
High		93.18%	94.32%
Low			95.57%
Sham			
**AUC of the ROC**			
**ReHo**			
High		0.72	0.84
Low			0.68
Sham			
**DC**			
High		0.73	0.82
Low			0.63
Sham			

*ReHo, regional homogeneity; DC, degree centrality; SVM, support vector machine; AUC of the ROC, the area under the curve of the receiver operating characteristic.*

**FIGURE 7:**
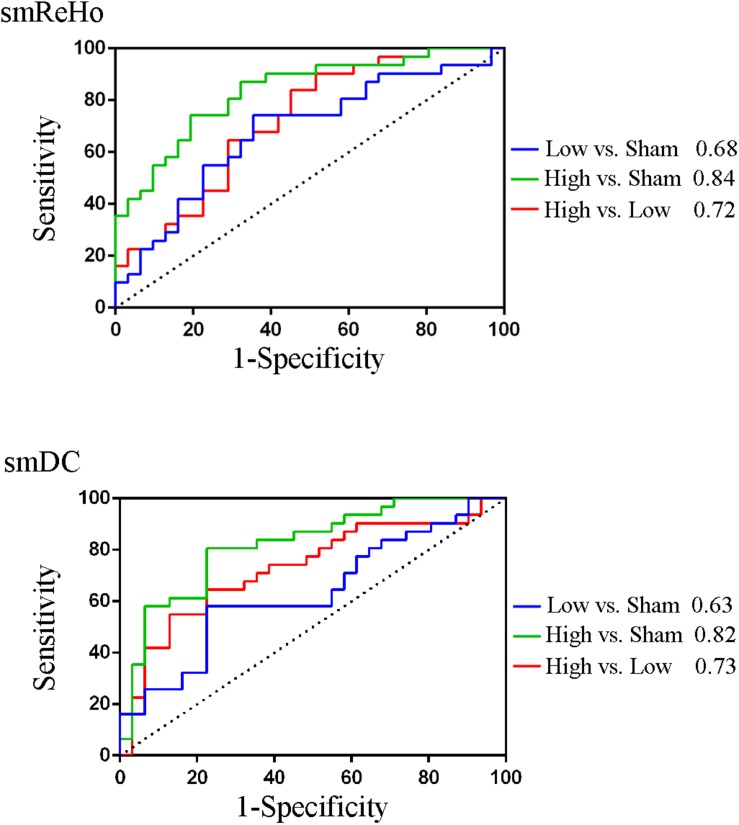
The area under the curve (AUC) of the receiver operating characteristic (ROC) of the peak voxels of the *F* maps of ReHo (*x* = 39, *y* = -60, *z* = -54) and DC (*x* = 42, *y* = -57, *z* = -54).

To compare against the SVM, we calculated the area under the curve (AUC) of the receiver operating characteristic (ROC) of the peak voxels of the *F* maps of ReHo (*x* = 39, *y* = −60, *z* = −54) and DC (*x* = 42, *y* = −57, *z* = −54), respectively. The pairwise comparisons showed 63–84% accuracy (Figure 7 and Table 4). As expected, the results were similar to the results of the pairwise *t* tests (Figures 5, 6 and Table 3). The comparison of ReHo and DC values for the high-frequency (10 Hz) condition vs. sham condition showed the most significant difference in the right cerebellum.

## Discussion

### Effects of rTMS in the Cerebellum

The motor cortex is a widely used stimulation target for rTMS (Lefaucheur, 2019). Many studies have claimed that FC alters after rTMS on the motor cortex (Nettekoven et al., 2014; Esterman et al., 2017; Ji et al., 2017; Hawco et al., 2018; Riedel et al., 2019; Shang et al., 2019). The seed selection and candidate networks varied among these studies. Although these findings are helpful for understanding the mechanisms of rTMS modulation, these results are less helpful to precisely localize the changes in activity, and hence are difficult to translate into clinical practice as they do not provide a precisely focused target for brain stimulation. We found significant condition effects of rTMS on local connectivity (ReHo) and global connectivity (DC) in areas VIII/VIIb of the right cerebellum (ipsilateral to the finger movement) in healthy participants. The cerebellum is involved in motor function via the cerebello-thalamo-cortical circuit (Middleton and Strick, 2001). The VIIb region receives projections from neurons in the subthalamic nucleus and input from the contralateral premotor areas (Wu and Hallett, 2013). The current rTMS study stimulated the left motor activation area and found significantly increased ReHo and DC in the right cerebellum in the high-frequency (10 Hz) condition, but not in the low-frequency or sham conditions (Figures 3, 4). The motor cortex has been commonly considered a stimulation target of rTMS treatment of movement disorders (Wagle Shukla et al., 2016). The current finding may therefore have direct importance for the rTMS treatment of movement disorders with dysfunction of the motor-thalamo-cerebellum circuit.

### The Classification Accuracy of the PAIR Method for SVM

It has been reported that the PAIR SVM method performs better than the conventional UNPAIR method in generalization to a completely new dataset in a within-group design (Zhou et al., 2017). We found the discriminative accuracy to be 93–97% (Table 4), with no apparent difference in accuracy across pairwise comparisons or across metrics (ReHo vs. DC). The spatial pattern of the weighted contribution identified by the classification was very similar to that of the paired *t* test maps of pairwise comparison between rTMS conditions (Figures 5, 6). In contrast to the high accuracy of SVM, no any single voxel could correctly differentiate between rTMS conditions, with the highest showing accuracy of only 84% (AUC of the ROC, Table 4). The SVM results suggest that both high- and low-frequency rTMS significantly modulated brain activity in widespread but distinct ways. Although only the right cerebellum survived correction for multiple comparisons, other motor-related regions including the left cerebellum and the bilateral sensorimotor cortices were also modulated by high- and low-frequency rTMS (Figures 5, 6). Although the effect size of these brain regions was small, combinations of these brain regions through the SVM could accurately differentiate between rTMS conditions.

### The Metrics ReHo and DC

Regional homogeneity reflects the temporal local synchronization or local connectivity of a given voxel with its nearest neighbors (7, 19, or 27 voxels) (Zang et al., 2004). DC calculates the total number of connections or total weighted connectivity of a given voxel with all other voxels in the brain (Buckner et al., 2009). ReHo has been reported to be decreased in the cerebellum in patients with movement disorders (Jiang et al., 2016; Liu et al., 2017). The current findings demonstrate that the ReHo value in the right cerebellar regions VIII/VIIb can be significantly enhanced by high-frequency rTMS in healthy participants, but not in low-frequency or sham stimulations. Although the patterns of DC and ReHo were very similar (Figure 2 upper vs. lower; Figure 5 vs. Figure 6), they did show slight discrepancies. Future studies should further compare the two methods.

### Distinct Effects of High- and Low-Frequency rTMS

A recently published review supported the viewpoint that high-frequency rTMS will recruit more neural networks than low-frequency rTMS (Lefaucheur, 2019). Since TMS activates circuits, the neurobiological changes in activity can be observed at areas distant from the stimulation site. For example, stimulating the precentral gyrus contralateral to a source of pain at a frequency of 5–20 Hz induces analgesic effects (Lefaucheur, 2016). Low-frequency (≤1 Hz) and high-frequency (≥5 Hz) stimulation are two classic rTMS paradigms and cause inhibitory effects through long-term depression (LTD) of synaptic transmission, and excitatory effects through long-term potentiation (LTP), respectively (Chen et al., 1997; Pascual-Leone et al., 1998; Post et al., 1999). Low-frequency pulsed TMS reduces the amplitude of MEPs, while high-frequency pulsed TMS enhances MEP amplitude (Lefaucheur, 2019). However, there is no evidence to assume from the MEP amplitude that inhibitory/excitatory effects are due to the LTD/LTP in other rTMS applications, such as rTMS treatment (Lefaucheur, 2019). The rTMS aftereffect highly depends on the property of the brain network and the status of the population (Lefaucheur, 2019). This makes it difficult to say whether low-frequency rTMS induces inhibitory effects, or high-frequency rTMS induces excitatory effects on brain function in remote regions. For instance, increased FC between the left posterior inferior parietal lobule and hippocampal formation was reported after low-frequency rTMS, and decreased FC between default mode network nodes has been reported after high-frequency rTMS (Eldaief et al., 2011). A similar paradox occurs with theta burst stimulation (TBS), another rTMS technique. Intermittent TBS (iTBS) is thought to be excitatory, and continuous TBS (cTBS) is thought to be inhibitory (Rossini et al., 2015). Nevertheless, increased FC was found after stimulation of the prefrontal cortex by cTBS in healthy controls (Dan et al., 2016), and decreased FC has been reported after stimulation of the parieto-occipital vertex by iTBS in patients after stroke (Volz et al., 2016).

The therapeutic importance of rTMS results from the modulatory effects it can mediate on certain abnormal brain areas. The results of the present study provide evidence for one such rTMS paradigm that has these effects: a total of 1800 10-Hz rTMS pulses successfully enhanced the ReHo and DC value in the cerebellum VIII/VIIb. The potential clinical significance is that high-frequency rTMS of the motor cortex could be applied to patients with movement disorders whose pathology is characterized by decreased ReHo or DC in the cerebellum. We also show that self-initiated finger movements are a useful task for individualized target localization. Multi-session rTMS could induce more reinforced and prolonged aftereffects as 5-Hz rTMS of the motor area for 2 weeks provides an improvement in clinical symptoms and increases the rate of return to normal low-frequency fluctuations (ALFF) (Liu et al., 2015). Nevertheless, evidence from neuroimaging studies can only provide us with suggestions for the modulatory effects of rTMS on brain function. Clinical improvement is the ultimate aim of rTMS interventions. More studies should be conducted to outline the underlying mechanisms involved.

## Conclusion

In conclusion, high-frequency (10 Hz) but not low-frequency (1 Hz) rTMS on the left motor cortex significantly increased ReHo and DC in the right cerebellum VIII/VIIb ipsilateral to the finger movement. SVM multivariate analysis showed 93–97% accuracy. Our results suggest that univariate and multivariate analysis, such as SVM, are mutually complementary. The univariate analysis could precisely localize the rTMS effect at a voxel level, illustrated by the finding that high- but not low-frequency rTMS enhanced ReHo and DC in the right cerebellum. Meanwhile, the multivariate analysis suggested that both high- and low-frequency rTMS significantly modulate brain activity in a widespread but distinct manner. Future studies should investigate which specific symptoms of movement-related disorders could be modulated by high- or low-frequency rTMS.

## Limitations

A few limitations of the current study should be addressed here. (1) It was a small sample study with a single session of stimulation. This could be the reason for the weak effect. (2) Although we used a stringent validation, the split-half test, the feature extraction step of the *F* map could be considered “double dipping” or circular analysis. Validation in a new dataset will provide a more reliable conclusion.

## Data Availability Statement

The datasets generated for this study are available on request to the corresponding author.

## Ethics Statement

The studies involving human participants were reviewed and approved by the Ethics Committee of the Center for Cognition and Brain Disorders (CCBD) at Hangzhou Normal University (HZNU). The patients/participants provided their written informed consent to participate in this study.

## Author Contributions

All authors: experimental design. X-PD, Z-JF, H-XW, YJ, and NZ: data collection. JW, X-PD, X-LL, and Y-YW: data analyses. JW, Y-FZ, and JZ: writing the manuscript.

## Conflict of Interest

The authors declare that the research was conducted in the absence of any commercial or financial relationships that could be construed as a potential conflict of interest.

The reviewer, HZ, declared a past co-authorship, with one of the authors, Y-FZ, to the handling editor.

## Footnotes

1 https://www.fil.ion.ucl.ac.uk/spm/software/spm12/

2 http://rfmri.org/dpabi

3 https://www.fil.ion.ucl.ac.uk/spm/software/spm12/

4 https://www.ibm.com/analytics/spss-statistics-software
